# Segregation-to-integration transformation model of memory evolution

**DOI:** 10.1162/netn_a_00415

**Published:** 2024-12-10

**Authors:** Luz Bavassi, Lluís Fuentemilla

**Affiliations:** Laboratorio de Neurociencias de la Memoria, IFIByNE - UBA, CONICET, Buenos Aires, Argentina; Departamento de Física, Universidad de Buenos Aires, Buenos Aires, Argentina; Department of Cognition, Development and Education Psychology, University of Barcelona, Barcelona, Spain; Institute of Neuroscience (UBNeuro), University of Barcelona, Barcelona, Spain; Bellvitge Institute for Biomedical Research, Hospitalet de Llobregat, Barcelona, Spain

**Keywords:** Neural network, Modularity, Memory, Consolidation, Reactivation, Plasticity

## Abstract

Memories are thought to use coding schemes that dynamically adjust their representational structure to maximize both persistence and efficiency. However, the nature of these coding scheme adjustments and their impact on the temporal evolution of memory after initial encoding is unclear. Here, we introduce the Segregation-to-Integration Transformation (SIT) model, a network formalization that offers a unified account of how the representational structure of a memory is transformed over time. The SIT model asserts that memories initially adopt a highly modular or segregated network structure, functioning as an optimal storage buffer by balancing protection from disruptions and accommodating substantial information. Over time, a repeated combination of neural network reactivations involving activation spreading and synaptic plasticity transforms the initial modular structure into an integrated memory form, facilitating intercommunity spreading and fostering generalization. The SIT model identifies a nonlinear or inverted U-shaped function in memory evolution where memories are most susceptible to changing their representation. This time window, located early during the transformation, is a consequence of the memory’s structural configuration, where the activation diffusion across the network is maximized.

## Author Summary

The Segregation-to-Integration Transformation (SIT) model provides a framework for memory transformation based on changes in the neural network properties. SIT posits that memories shift from highly modular to less modular network forms over time, driven by neural reactivations, activation spread, and plasticity rules. The SIT model identified a critical period, shortly after memory formation, where reactivations can induce significant structural modifications. As repeated reactivation passes, the network becomes more stable and integrated, becoming more resistant to change, thus preserving the core information while reducing the likelihood of distortion or loss.

## INTRODUCTION

Memories, like other natural systems, evolve to enhance survival in organisms. They adapt their representational structures to meet experiential needs better, aiming for optimized persistence and efficacy. While there is an agreement that memory evolution involves representational changes, the balance between persistence and efficacy is still not fully understood. A major research challenge is clarifying how experiences that enhance efficacy influence memory changes, ultimately promoting persistence.

Our current understanding of memory evolution suggests that, initially, memories are encoded by groups of neurons with synchronized activity, forming clustered neural ensembles that, if reactivated, induce memory retrieval (Josselyn & Tonegawa, 2020). These neural ensembles are identified in the hippocampus, but over time, they undergo structural changes, losing their modular properties in the hippocampus (Gonzalez, Zhang, Harutyunyan, & Lois, 2019), and other neural ensembles beyond the hippocampus gradually assume their representation (Frankland & Bontempi, 2005). This transition, however, occurs relatively slowly and requires repeated reactivation of the neural ensembles to shift the structure of the neural ensembles between networked regions (Frankland & Bontempi, 2005). For a while, the dominating view was that once memory neural ensembles shifted beyond the hippocampus, they became consolidated and stable in the long term. More contemporary views advocate that consolidation and reactivation may act in the service of generalization (Sun, Advani, Spruston, Saxe, & Fitzgerald, 2023). That is, given that individual memorized experiences rarely repeat exactly, generalization allows us to identify systematic relationships between features of the world, ultimately involving extending learned information to novel contexts. Thus, generalization involves linking and extracting commonalities among various memories. As the brain generalizes, initial memory representations transform their structure, becoming intricately connected to related memories (Nadel & Moscovitch, 1997; Winocur, Moscovitch, & Bontempi, 2010). Through this interconnected web of associations, the initially clustered memory representations become part of a broader network, where each memory influences and is influenced by others. These processes ensure that consolidated memories are not isolated but integrated into an interconnected form, making them applicable to various situations and contributing to the adaptive evolution of memories (Shohamy & Wagner, 2008; Sutherland & Lehmann, 2011). Thus, while most extant views of memory evolution offer insights into memory reorganization and generalization, developing a formal model that encapsulates the dynamic restructuring of memory networks remains an open area of research. Such a model would advance our understanding of memory consolidation, generalization, and the complex associations involved in adaptive memory evolution.

**Figure 1.**
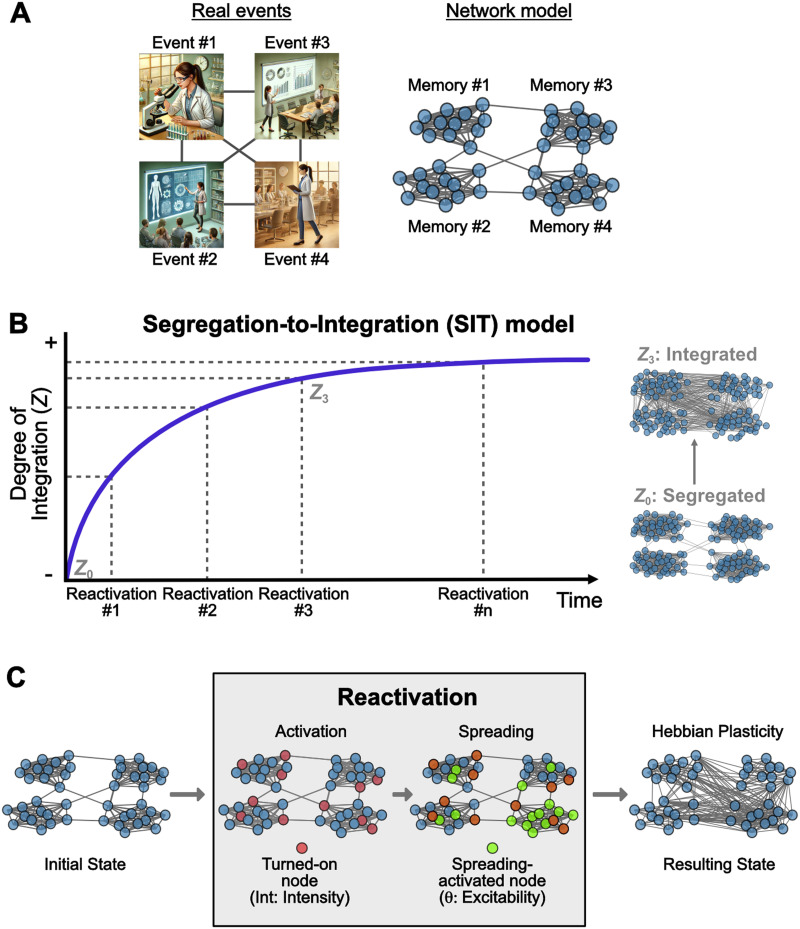
Schematic depiction of the SIT model. (A) The representation of memory as networks. Four separate experiences sharing a common element, for example, research activities, are represented by four subgroups of nodes tightly connected within them and sparsely linked with the nodes of the rest of the network. (B) The central tenet of the SIT model is that memory networks tend to shift from highly segregated to an integrated configuration guided by repeated reactivations over time. The Degree of Integration (*Z*) reflects the structural properties of the network, *Z* ∼ 0 implies a network strongly segregated, whereas *Z* ∼ 1 represents a network with a more integrated structure. (C) Memory reactivation induces retrieval and reorganization of the memory network. An external input that overlaps with the original experience triggers memory reactivation, and a rate of nodes of the memory network is turned on (*Int*), transitioning its mode to an active state. These turned-on nodes initialize the propagation of the activation over the network. The extent of the spreading is controlled by the excitability threshold parameter *θ*. Once the network achieves the steady state, a Hebbian plasticity rule modifies the connectivity pattern, creating and removing links between nodes.

We propose the Segregation-to-Integration Transformation (SIT) model to account for how memory representations are transformed via repeated reactivations over time. SIT posits that memories are represented as neural network ensembles, with neurons (nodes) linked by connections (edges), and that memory transformation occurs through changes in the connectivity patterns between these ensembles (Ortega-de San Luis, Pezzoli, Urrieta, & Ryan, 2023; Ryan, Ortega-de San Luis, Pezzoli, & Sen, 2021; Ryan, Roy, Pignatelli, Arons, & Tonegawa, 2015; Tonegawa, Pignatelli, Roy, & Ryan, 2015). The SIT model assumes that memory networks are initially represented by information of single events encoded by groups of tightly connected neurons, sparsely linked with other neuronal groups, that represent other episodes with a common element or feature. This common element may be a concept, a temporal window, or a causal relationship (de Sousa, Chowdhury, & Silva, 2021). Highly modular networks serve as buffers to contain activation within their originating community rather than spreading to other communities (M. E. J. Newman, 2006). This modularity enhances storage capacity (Brunel, 2016) and represents an optimized configuration for early memories that balances the need to shield against disruptions while accommodating substantial information storage (Gonzalez et al., 2019). For example, the memory of a day at work may include memories of four different event experiences: working on a specific project, giving a lab talk, attending a lab meeting, and lecturing at the university (Figure 1A). While memories for these specific events can be stored and retrieved independently, after repeated encounters of similar experiences, they may become intricated into a broader memory structure that gives rise to an integrated form representing the experience of “my research activities.” We proposed that, over time, repeated reactivations transform the highly modular network structure into a more integrated form, incorporating links to nodes that represent overlapping information. This transition to a lower modular structure facilitates memory retrieval (Watrous, Tandon, Conner, Pieters, & Ekstrom, 2013; Westphal, Wang, & Rissman, 2017), intercommunity spreading, and promoting generalization. By investigating the dynamic coding schemes of memories, we seek to elucidate the mechanisms by which memories adapt to enhance survival and optimize functionality over time.

## RESULTS

The SIT model seeks to analyze the evolution of memory within the context of network dynamics. SIT model focuses on the spatial organization of the network to represent memory accessibility, defined by how activation spreads through the network when specific nodes are activated. We propose that the initial memory state is represented by a set of nodes exhibiting a highly segregated structure that gradually shifts toward an integrated form induced by repeated reactivations (Figure 1B). In a segregated network, activation is more likely to remain confined within specific clusters or communities, leading to a more context-specific and isolated recall. Conversely, in a highly integrated network, activation can spread more easily and efficiently across the network, facilitating the retrieval of interconnected memory components. This means that a memory cue can activate a broader, more cohesive set of related memories, enhancing holistic recall. The Degree of Integration, therefore, reflects the memory network’s structural configuration and its functional capacity to support different types of memory retrieval processes.

The SIT model posits that memory transformation is represented by changes in network structure, which depends on a shift from a segregated to an integrated form. Analytically, this can be assessed by quantifying the number of links between communities relative to the total number of possible links in the network. We labeled the output of this computation as the Degree of Integration (*Z*). *Z* ∼ 0 implies a network strongly segregated, whereas *Z* ∼ 1 yields more links between communities, that is, a more integrated structure (Figure 1B). Thus, we can study the segregation-to-integration dynamics over time by computing it for each resulting network after a reactivation.

We modeled memory reactivation following a deterministic rule that can induce memory network reorganization (Figure 1C). An external input or cue that overlaps with the original experience triggers memory reactivation, and a rate of nodes of the memory network is turned on (named as intensity, *Int*), transitioning its mode from an inactive to an active state (from 0 → 1). We assumed that a larger overlap between the current input and the existing memory would increase the reactivation’s intensity (*Int*) (Wammes, Norman, & Turk-Browne, 2022). The nodes turned on initialize the propagation of the activation over the network based on a Diffusion Linear Model (Nematzadeh, Ferrara, Flammini, & Ahn, 2014). Although it has been effective in capturing recurrent information transmission and retention in social networks (Centola, 2010; Danon, Arenas, & Díaz-Guilera, 2008), lately, it was proposed as a possible mechanism of brain network communication, both in the micro- and macroscopic scale. The Diffusion Linear Model included an excitability threshold parameter *θ* to control the extent of the propagation. We conceived this threshold parameter as a proxy for the neuronal excitability state of network nodes. By systematically varying its value in our model, we assessed whether fluctuations in neuronal excitability influence memory ensemble interactions, promoting either integration or separation (Josselyn & Frankland, 2018). Finally, we implemented a plasticity rule that modifies the connectivity pattern, linking active nodes to promote pairwise activity, similar to Hebbian plasticity (Hebb, 2005). Additionally, we included a weakening rule that removes connections between active and inactive nodes (Stent, 1973).

**Figure 2.**
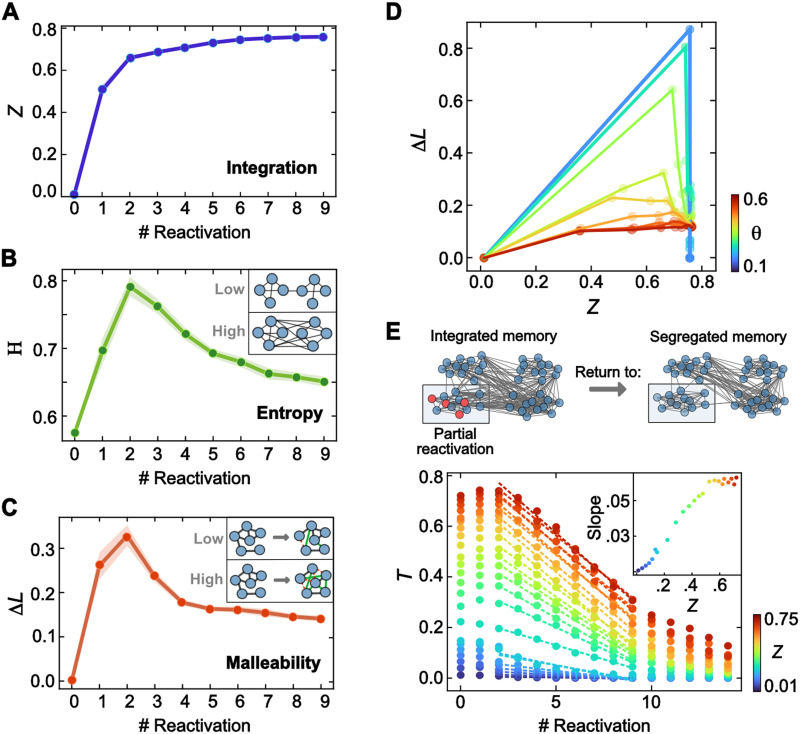
Model simulations. (A) Evolution of the Degree of Integration (*Z*) over 10 repeated reactivations. *Z* increases monotonically up to a highly integrated configuration. (B) Network entropy’s evolution (*H*) over repeated reactivations. All the networks present high entropy levels, reaching the maximum early when the network is not completely integrated. (C) Malleability index (Δ*L*) over reactivations. Δ*L* presents a similar pattern to entropy. Intensity (*Int* = 0.3 per community), the excitability parameter (*θ* = 0.4), and the initial structural configuration (*Z*_0_ = 0.01) were fixed for the reported data in panels A, B, and C. (D) Relationship among *Z* and Δ*L* for different excitability thresholds for fixied intensity (*Int* = 0.3). The green trajectory is the conjunction of panels A and C. (E) The tightness index evolution over 15 partial repeated reactivations (only one community was reactivated with fixed intensity, *Int* = 0.3). Colors represent the initial network’s Degree of Integration (*Z*). The excitability parameter was fixed at 0.4. The inset panel is the linear regression slope of tightness over reactivation numbers 3 and 9. The data reported are an average of multiple experimental runs (25) where the identity of the turned-on nodes is randomized in each run.

To investigate memory transformation, we prepared an ensemble of highly segregated networks (*Z* = 0.01) with 128 nodes, divided equally into four communities of 32 nodes each. We then simulated reactivation by randomly activating 30% of each community’s nodes (*Int* = 0.3) with a fixed excitability threshold at *θ* = 0.4. We studied how each reactivation induced changes in the memory network by computing the Degree of Integration (*Z*). In line with the SIT hypothesis, the results of this analysis revealed that *Z* increased monotonically due to repeated reactivations, confirming that reactivating overlapping nodes effectively shifted the original segregated network memory configuration into an integrated network form in our model. We also found that this configural network change occurred rapidly within the first four reactivations, after which *Z* increased minimally with further reactivations (Figure 2A). These findings indicate that the mathematical rules used to simulate reactivation align with the central tenet of the SIT model: Memory undergoes a one-way transformation from a segregated form to an integrated configuration.

Network shifts from a segregated to an integration form should entail changes in how the spreading is distributed through the network along the process. Therefore, changes in the spatial structure of the network may be a determinant in modulating memory accessibility during its evolution. We examined this issue by computing network entropy (*H*). Entropy measures the information flow over a network based on the probability of jumping between nodes when traveling randomly through a network (Wiedermann, Donges, Kurths, & Donner, 2017). Sparse network configurations involve less entropy (i.e., *H* ∼ 0) as there is low uncertainty along which path the information is transferred upon activation. In contrast, fully connected network configurations involve high entropy (*H* ∼ 1), as any activated node has high uncertainty in the activation spread. In the context of the SIT model, we found that entropy was relatively high (i.e., *H* > 0.5) in all stages of network evolution (Figure 2B). However, the entropy curve peaked around the second reactivation, indicating the highest efficiency in information transmission at this point. This peak occurred before the network became highly integrated, as described in our previous analysis, thereby suggesting the existence of a critical time window during memory evolution at which a reactivation can access a larger portion of the stored memory.

The findings of maximum activation transfer at early stages of memory evolution indicate the existence of an optimal stage when network memory is most accessible as a whole. This suggests the existence of a specific stage in memory evolution where reactivation may induce greater changes in connectivity patterns, resulting in a higher degree of malleability. To account for this possibility, we computed the Malleability Index (Δ*L*; Figure 2C), which quantifies changes in the number of connections within the network. The Malleability Index is expected to be low if a reactivation induces minimal changes to the existing network connectivity configuration, whereas it would be high if a reactivation elicits substantial changes to the existing number of connections. In the context of SIT, we expect low Malleability Index values when the memory network reaches a stable integrated form (i.e., during later reactivation stages). In contrast, we expect higher Malleability Index values at the early stages of memory transformation, aligning with the previously observed entropy function. This analysis revealed that the Δ*L* values evolved nonlinearly throughout memory transformation, displaying an inverted U-shaped function. Initial reactivations induced substantial changes in the connectivity pattern of the memory networks, reaching a maximum Δ*L* at around four to five reactivations. However, after achieving the maximum, Δ*L* decreased and flattened around a low level above 0. This suggests the existence of a specific stage in memory evolution where reactivation may induce greater changes in the wire patterns, resulting in a higher degree of malleability, and implies that once this malleability window has passed, reactivation still induces memory changes, albeit at a minimal level.

The low-dimensional reactivation proposed in the SIT model involves a unique parameter, the excitability threshold (*θ*). It is an intrinsic parameter of spreading, as it limits the degree of activation throughout the network. We explored the extent to which the SIT findings depended on variation on this parameter in a network by calculating the trajectories of memory evolution in the *Z* − Δ*L* space for different excitability thresholds *θ* (Figure 2D). The nonlinear relation between *Z* and Δ*L* is displayed as an open loop in the phase space (green line; *θ* = 0.4). As expected, the amplitude of the nonlinearity increases for more permissive thresholds. That is, lower excitability thresholds favored the spreading of activation. After the maximum malleability occurred, memories involving more permissive excitability thresholds did not present changes in the number of connections (lack of malleability). In late reactivations, memories with stringent thresholds showed limited variability in the Malleability Index. Overall, the excitability threshold modulated the memory’s malleability during the early phase of the process and constrained the potential for change in an integrated memory.

While the results of the SIT model revealed a one-way tendency of network memories to shift from a segregated to an integrated form, sometimes, preserving a discrete representation of memory over time is necessary. Theoretical models have shown that this can be achieved through pattern separation mechanisms that encode an orthogonal representation of the original memory (Favila, Chanales, & Kuhl, 2016; O’Reilly & Norman, 2002; Treves & Rolls, 1994) or by reinforcing the memory individually through repeated reactivation (Lohnas et al., 2018). We, therefore, asked whether a network’s community can remain discrete and identifiable within the network upon its reactivation. To address this, we implemented the repeated reactivation of 30% of nodes from one original memory (one community), even at states where the network structure has already reached a relatively integrated form (Figure 2E). We defined the tightness index (*T*) as a measure of the strength of the community (the number of links outside the community over the total number of links). *T* ∼ 0 implies almost null links outside the community, implying an isolated community; *T* ∼ $\frac{1}{2}$ implies the same number of links within the community as to the rest of the network, while a community with a small number of internal links achieves *T* ∼ 1. This served as a proxy to determine how well the memory returned to a segregated form or how it resisted integration. This analysis revealed that the *T* value decreased for all *Z* states of the original network, reaching a low value (i.e., a highly segregated network) after several reactivations. These findings indicate that even when the original network was in highly integrated states, the repeated reactivation of partial elements from one of the four communities could return the network to a segregated form, irrespective of its original state of integration. However, when we measured the slope of the decreasing *T* function, we found that the speed of this returning function varied nonmonotonically throughout the *Z* state. Specifically, *T* slowed its rate of decrease linearly from low *Z* to intermediate *Z* states (approximately 0.6), upon which it reached a similar decrease function (inset Figure 2E). The model shows that reactivation requires elements of different communities to achieve the integration process. If reactivation involves elements of only one community, the memory becomes isolated, remaining protected.

So far, our findings have been based on a fixed network size (i.e., *N* = 128) and fixed reactivation strength (i.e., *Int* = 0.3). While these parameters are set arbitrarily in our modeling, they may influence the temporal dynamics of network transformation. Therefore, an open question that remains to be explored is whether our results can be generalized when these parameters are varied across their full range of values.

**Figure 3.**
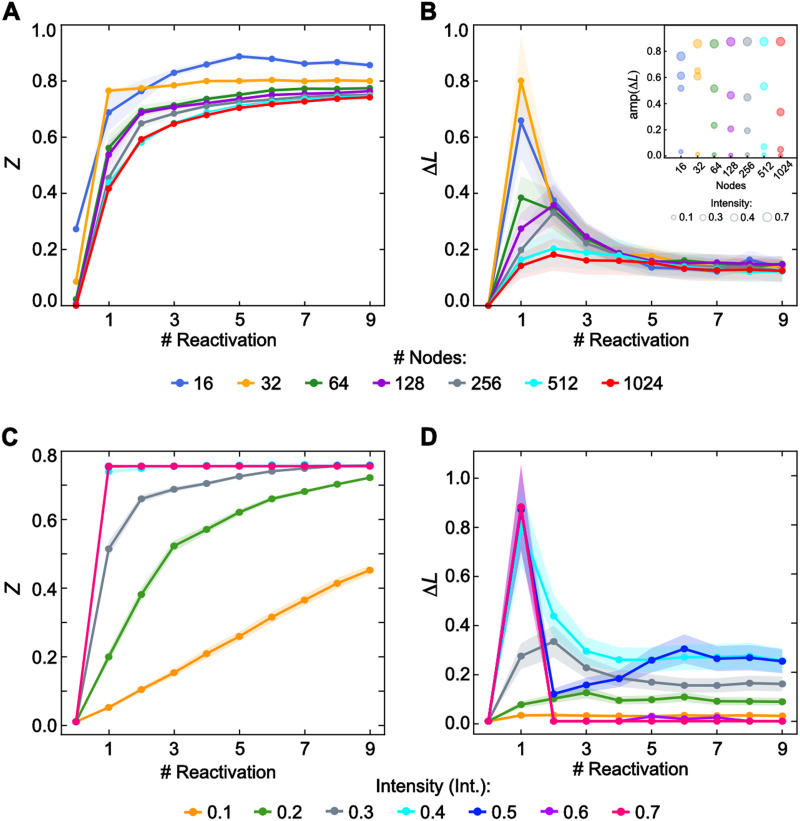
Model generalization. (A) Degree of Integration and (B) Malleability Index over 10 repeated reactivations for different network’s sizes (*N* = 16, 32, 128, 256, 512, 1024). Intensity (*Int* = 0.3) and excitability (*θ* = 0.4) were fixed. The inset panel in B depicted the maximum of malleability for different network sizes, calculated by subtracting the peak value of the function from the averaged value of the five last values. The size of the circles represented the intensity level (*θ* = 0.4). (C) Degree of Integration and (D) Malleability Index over 10 repeated reactivations for different intensities. The networks had 128 nodes and four communities; the excitability parameter was fixed at *θ* = 0.4. The data reported are an average of multiple experimental runs (25) where the identity of the turned-on nodes is randomized in each run.

First, we tested that the memory transformation described did not depend on the size of the network by expanding our analysis to include networks of various sizes (for fixed *Int* = 0.3 and *θ* = 0.4). We analyzed the dynamics of the Degree of Integration (*Z*) and the Malleability Index (Δ*L*) for highly segregated networks consisting of 16, 32, 64, 128, 256, 512, and 1,024 nodes (Figure 3A and B). Each network was divided into four equal communities with different initial Degree of Integration (*Z*_16_ = 0.3; *Z*_32_ = 0.09; *Z*_64_ = 0.02; *Z*_128_ = 0.01; *Z*_256_ = 0.001; *Z*_512_ = 0.0004; *Z*_1024_ = 0.0001). The Degree of Integration (*Z*) exhibited similar behavior across memory networks of different sizes, showing a monotonic increase in *Z* during reactivations and reaching a fully integrated state early on. The speed of integration did not appear to depend on the network size, although the smallest networks achieved a more integrated configuration. However, the curves of malleability displayed different profiles for networks of different sizes. The inverted U-shaped function was observed only in small networks. In contrast, larger networks exhibited a monotonic increase in the malleability function, probably because the nonlinearity rose with a different set of parameters (more permissive excitability thresholds or higher intensity values). To disentangle this, we depicted the maximum amplitude of malleability for different network sizes and intensity levels (with a fixed excitability threshold *θ* = 0.4; inset Figure 3B). As expected, the U-shaped function appeared for all network sizes, but larger networks need higher intensities to display the nonlinearity. Interestingly, all the networks showed the same level of malleability after achieving their maximum.

Finally, we analyzed the role of intensity in our findings. Intensity (*Int*) refers to the rate at which network nodes are activated in each reactivation. Figure 3C and D display the Degree of Integration (*Z*) and Malleability Index (Δ*L*) for a network of four communities (32 nodes per community) with a fixed excitability threshold (*θ* = 0.4). Starting with a segregated configuration (*Z* = 0.01), networks reactivated with the highest intensities achieved a highly integrated state after the first activation. In contrast, the lowest intensities induced a slow integration process, with networks reactivated at *Int* = 0.1, failing to achieve an integrated configuration even after 10 reactivations, requiring additional activations to reach the final state. The Malleability Index (Δ*L*) exhibited a U-shaped function for all *Int* > 0.2. Notably, networks reactivated with the highest intensities showed no changes in malleability after reaching the maximum.

In summary, the SIT model is robust, extending to memories of different sizes and reactivations with varying intensity strengths.

## DISCUSSION

The SIT model provides a framework for understanding how memory evolves, summarized through changes in neural network properties. SIT posits that memories shift from highly modular to less modular network forms over time, driven by neural reactivations, activation spread, and plasticity rules. The SIT model identified an optimal window of memory malleability, indicating a nonlinear, inverted U-shaped function, where memories are most malleable early on and gradually become less changeable. This suggests the existence of a critical period shortly after memory formation, during which the memory network is highly plastic and susceptible to changes. During this period, reactivations can induce significant structural modifications, allowing for the integration of new information and the strengthening or weakening of existing connections. As time progresses and the memory undergoes repeated reactivations, the network becomes more stable and integrated, leading to a decrease in malleability. This stabilization process implies that the memory is becoming more resistant to change, thus preserving the core information while reducing the likelihood of distortion or loss.

An underlying assumption of the SIT model is that the transformation of memory relies on modifications in connectivity patterns among nodes within the memory network. This perspective aligns with the idea that learning involves modifications to the wiring diagram of a neural ensemble, where previously unconnected units establish connections and vice versa (Ortega-de San Luis et al., 2023; Ryan et al., 2015, 2021; Tonegawa et al., 2015). However, while alterations in the wiring pattern offer a potential substrate for encoding more extensive information in sparse coding models (Chklovskii, Mel, & Svoboda, 2004; Knoblauch, Palm, & Sommer, 2010), it also underscores that other forms of plasticity based on changes in synaptic weights, the strength of connections between cells, are crucial for comprehending memory evolution (Bonhoeffer & Yuste, 2002; Chklovskii et al., 2004). Although both mechanisms are likely involved in engram cell formation and function (Poo et al., 2016), investigations of network properties in hippocampal code representation have shown that wiring diagrams effectively encode specific experiences (Ortega-de San Luis et al., 2023; Ryan et al., 2021). In addition, shifts in these connectivity patterns provide a more robust explanation for memory transformation than alterations in the individual firing properties of the neurons (Gava et al., 2021). In subsequent work, the simplicity of the model may enable us to readily incorporate new variables that account for changes in synaptic weights encompassing both modifications in the wiring diagram and the strength of the connections.

The SIT model identified an optimal window of memory malleability, where memories are most malleable early on and gradually become less changeable. This result may reconcile theoretical models of memory consolidation (McClelland, McNaughton, & O’Reilly, 1995; Nadel & Moscovitch, 1997; Squire, 2004; Winocur & Moscovitch, 2011) and reconsolidation (Nader & Hardt, 2009; Sara, 2000), which both suggest that memories change over time. However, while reconsolidation posits one-shot changes, consolidation highlights continuous evolution (Dudai & Eisenberg, 2004). Our model shows that memories can evolve perpetually but have an optimal window of malleability early on. This aligns with the idea that young memories are more susceptible to disruption, while older ones are more stable (Alberini, 2011; Eisenberg & Dudai, 2004; Frankland & Bontempi, 2005; Milekic & Alberini, 2002; Suzuki et al., 2004). After this window, memories can still change, but minimally (McKenzie & Eichenbaum, 2011; Roüast & Schönauer, 2023).

We conceptualized that the network’s excitability threshold and the reactivation’s intensity modulated structural changes in the memory network. The excitability threshold was included to reflect findings from rodent studies showing that memory allocation depends on the intrinsic excitability of cells (Josselyn & Frankland, 2018). Fluctuations in neuronal excitability dictate how engrams interact, promoting either memory integration or separation. The intensity of reactivation manipulated neuron coactivation based on the overlap between the current input and existing memory (Wammes et al., 2022). Recent theoretical models, like the nonmonotonic plasticity hypothesis, suggest a U-shaped pattern of representational change: Low coactivation results in no change, high coactivation strengthens connections, and moderate coactivation leads to differentiation (Detre, Natarajan, Gershman, & Norman, 2013; E. L. Newman & Norman, 2010; Ritvo, Turk-Browne, & Norman, 2019). Our model found that excitability and intensity affected the rate of memory transition from segregated to integrated forms depending on the network size. Higher excitability and intensity increased propagation and memory malleability, while lower levels restricted activation spread and decreased malleability. Intermediate levels produced distinct malleability peaks, with maximum malleability occurring when the memory configuration was neither very segregated nor fully integrated, aligning with the nonmonotonic plasticity hypothesis.

The SIT model proposes that the shift of memories from segregated to integrated forms responds to the adaptation of the memory system to highly dynamic environments, where novel and past experiences may only partially overlap, necessitating a balanced interplay between stored information and accessibility. In this context, highly segregated memories may function suboptimally, owing to their limited accessibility (Hintze & Adami, 2008). In contrast, highly integrated memory network forms prioritize accessibility over storage quality, offering an adaptive structural configuration in ever-changing contexts. Network science has shown that at the intersection of these two extreme network configurations, there exists an optimal modularity configuration that effectively balances memory storage and information diffusion (Nematzadeh et al., 2014; Rodriguez, Izquierdo, & Ahn, 2019). Similarly, we propose that such an optimal modularity structural state of a memory form is ideal for inducing changes because it provides a balanced trade-off between the probability of being reactivated and the diffusion of activation throughout the network.

While the SIT model provides a low-dimensional framework in which memory is proactively shaped into a generalized form that involves an integrated state, it fails to address important issues. One such open question pertains to the distribution of network configuration and how the network configuration and its transformation are distributed in the brain. Another remaining issue would be to study the effects of memory reactivation under different brain states, such as wake and sleep stages, that are thought to determine the nature of memory transformation. For example, reduced excitability and synaptic downscaling are thought to occur during the Slow Wave Sleep stage, but increased reactivation intensity and synaptic potentiation may better take place during the rapid eye movement (REM) sleep stage. While we await a better characterization of these issues, our model offers a simple guiding principle based on the topological structure of neural networks by which memories transform over time.

## MATERIALS AND METHODS

Simulations were done using homemade Python codes, mainly using Networkx (https://networkx.org/; a package for the creation, manipulation, and study of the structure, dynamics, and functions of complex networks).

### Building Networks

The Segregated-to-Integrated Model studied the evolution of a set of one-layer, unweighted, and undirected networks. Each node could achieve one of two possible values: *s* = {0, 1}, where 1 represented the “active” mode and 0 was the “inactive” one.

Initially (*T* = 0), the networks have *N* = {16, 32, 128, 256, 512, 1024} nodes split into *C* = 4 equal communities of *N*/*C* nodes each. Each community is a random regular graph, where each node has the same number of neighbors. We then linked the communities with *L^ext^* edges assigned randomly to pairs of nodes in different communities. This arrangement ensured that every node and community was not isolated and that they played a similar role in the network.

The mean node degree within the community was set to *k*_*int*_ = $\frac{1}{2}$*N*/*C* and the number of edges that connected different communities varies from 1 to *N* − 1. To formalize the state of the network, we quantified the Degree of Integration following Equation 1:

$$Z=\frac{1}{L}\sum_{i}^{C}\sum_{\begin{array}{c} n\in {𝒞}_{i}\\ m\notin {𝒞}_{i} \end{array}}L_{n,m}^{\mathrm{ext}}$$ (1)

where *L* is the total number of edges and $L_{n,m}^{\mathrm{ext}}$ are edges that linked node *n*, a member of the initial community 𝒞_*i*_, with node *m* that belongs to another community. Thus, *Z* ∼ 0 defines a highly segregated network, while *Z* ∼ 1 implies a network fully integrated (Figure 1B).

### Reactivation

Computationally, memory reactivation involves a sequence of three deterministic rules: First, we activate a random set of nodes (turn-on); second, we spread the activation throughout the network (spreading); and finally, we modify the connection pattern of the network (plasticity).

#### Turn-on.

The reactivation intensity (*Int*) is the fraction of inactive nodes that transition to the active state (from 0 → 1). It ranged from 0.1 to 0.7, with increments of 0.02. To introduce variability, the number of activated nodes per community was determined by sampling from a normal distribution with a mean of *Int* and a standard deviation of 0.05. To decide which nodes of the community become active, we used a uniform random number generator that selects values between 1 and *N*/*C*.

#### Spreading.

We adopted a Diffusion Linear Threshold model (Nematzadeh et al., 2014) to achieve activity propagation over the network. The state of node *n* is updated according to the following linear threshold rule, Equation 2:

$$S_{n}\left(t+1\right)=\left\{\begin{array}{ll} 1 & \text{if}\;\theta k_{n}<\sum_{j\in N_{n}}S_{j}\left(t\right)\\ 0 & \text{otherwise} \end{array}\right.$$ (2)

where *θ* is the excitability threshold parameter, *k*_*n*_ was the node degree, and *j* ∈ *N*_*n*_ represented the neighbors of node *n*. Throughout the simulations, *θ* varied between 0.1 and 0.6, with increments of 0.02. Once a node becomes active, it remains in this state. The spreading persisted until the network reaches a stable state, defined as *S*_*n*_(*t* + 1) = *S*_*n*_(*t*), for all *n* or exceeds 50 iterations (Nematzadeh et al., 2014).

#### Plasticity.

Once the network reaches a stable state (T), we implement a plasticity rule. This step modifies the connectivity matrix of the neural network by creating/removing an edge between two nodes *L*_*n*,*m*_ at *T* + 1. If both nodes are in an active state, they are connected, and the link between both is removed if the nodes are uncorrelated (one in an active mode and the other in an inactive state; Equation 3):

$$L_{n,m}\left(T+1\right)=\left\{\begin{array}{ll} 1 & \text{if}\;S_{n}\left(T\right)=S_{m}\left(T\right)=1\\ 0 & \text{if}\;S_{n}\left(T\right)\neq S_{m}\left(T\right)\\ L_{n,m}\left(T\right) & \text{otherwise} \end{array}\right.$$ (3)

### Memory Transformation

Memory transformation is simulated by applying a sequence of reactivations (10 or 15). The intensity (*Int*) remains constant throughout each reactivation. The nodes activated in each iteration were randomly assigned, as described above. We repeated the entire network transformation with the same set of parameters 25 times to characterize the mean behavior independently of the random assignment of nodes.

### Entropy

Network entropy is based on the classical Shannon entropy for discrete distributions (Freitas, Aquino, Ramos, Frery, & Rosso, 2019). It relies on the probability that a random walker goes from node *n* to any node *m*. The probability distribution *P*^(*n*)^ has entries *p*_*n*→*m*_ = $\frac{1}{k_{n}}$ (if *n* is connected with *m*) and 0 otherwise. So, the entropy per node is defined (Equation 4):

$$h_{n}\left(P^{\left(n\right)}\right)=-\sum_{m=1}^{N-1}p_{n\to m}\ln\left(p_{n\to m}\right)=\ln\left(k_{n}\right)$$ (4)

and *h*_*n*_(*P*^(*n*)^) = 0 if node *n* is fully disconnected.

Finally, the average normalized entropy, called the network entropy (*H*), is as follows (Equation 5):

$$H\left(P\right)=\frac{1}{N\ln\left(N-1\right)}\sum_{n=1}^{N}h\left(P^{\left(n\right)}\right)$$ (5)

### Malleability Index

To focus on the plasticity of memory, we study changes in the number of edges. The Malleability Index (Δ*L*) is the number of created (*L*^*c*^) and removed (*L*^*R*^) edges over the total number of edges before reactivation (*L*_−1_; Equation 6).

$$\Delta L=\frac{L^{c}+L^{R}}{L_{-1}}$$ (6)

Δ*L* is a positive parameter, and 0 value means no change in the number of edges.

To determine the existence of the inverted U-shaped function for networks of different sizes, we analyze the amplitude of the nonlinearity (substrating the final level of malleability) following Equation 7:

$$amp\left(\Delta L\right)=\text{max}\left\{\Delta L\right\}-<\Delta L{>}_{-5}$$ (7)

where < Δ*L* >_−5_ is the mean of malleability of the last five reactivations.

### Preserved Identity of a Community

To study how well a memory returned to a segregated form or how it resisted integration, we ran a sequence of 15 repeated reactivations for various networks of different Degree of Integration (*Z* between 0.001 and 0.6). The excitability threshold was *θ* = 0.4, and the networks had 128 nodes. Reactivation intensity was fixed as *Int* = 0.3, and it only involved nodes of one community (𝒞). We repeated each network transformation with the same set of parameters 25 times. For each reactivation, we calculated the strength or cohesion of the reactivated community (𝒞) with the tightness measure (*T*_𝒞_; Equation 8):

$$T_{𝒞}=\frac{1}{L_{𝒞}}\sum_{\begin{array}{c} i\in 𝒞\\ j\notin 𝒞 \end{array}}L_{i,j}^{\mathrm{ext}}$$ (8)

where *L*_𝒞_ is the total number of edges of the community *C* and $L_{i,j}^{\mathrm{ext}}$ are edges that linked node *i* (a member of the community 𝒞) with node *j* of the rest of the network. Thus, *T*_𝒞_ ∼ 0 defines a highly cohesive community while *T*_𝒞_ ∼ 1 implies a weak community, fully integrated with the rest of the network.

We analyze the slowed rate of *T* over the reactivations. We linearly fit the *T*′*s* evolution from reactivation number 3 to 9 and calculate the slope.

## ACKNOWLEDGMENTS

This work was supported by a grant from the Agencia Nacional de Promoción Cientfica y Tecnológica PICT 2020 - 00956 to L.B. and from the Spanish Ministerio de Ciencia, Innovación y Universidades, which is part of Agencia Estatal de Investigación (AEI), through the project PID2022 - 140426NB - I00 (funded by MCIN/AEI/10.13039/501100011033/ and FEDER a way to make Europe) to L.F. We thank CERCA Programme/Generalitat de Catalunya for institutional support.

## AUTHOR CONTRIBUTIONS

Luz Bavassi: Conceptualization; Data curation; Formal analysis; Methodology; Software; Visualization; Writing – original draft; Writing – review & editing. Lluis Fuentemilla: Conceptualization; Data curation; Investigation; Methodology; Project administration; Validation; Visualization; Writing – original draft; Writing – review & editing.

## FUNDING INFORMATION

Luz Bavassi, Fondo para la Investigación Científica y Tecnológica (https://dx.doi.org/10.13039/501100006668), Award ID: PICT 2020 - 00956. This work was supported by the Spanish Ministerio de Ciencia, Innovación y Universidades, which is part of Agencia Estatal de Investigación (AEI), through the projects PID2019-111199GB-I00 and PID2022-140426NB-I00 to L.F. (funded by MCIN/AEI/10.13039/501100011033/ and FEDER a way to make Europe).

## TECHNICAL TERMS

Modularity: An index of the degree to which network nodes are organized into discrete communities.
Segregated network: A network that has a high number of communities.
Integrated network: A network with a homogeneous connection distribution between its nodes.
Spatial structure of the network: The pattern of network connectivity, which describes the configuration of the wiring diagram.
Malleability: Changes in the number of connections or links of the network.
